# Anomalous Cervical External Carotid Artery-Internal Carotid Artery Anastomosis Diagnosed Using Digital Subtraction Angiography: A Case Report

**DOI:** 10.7759/cureus.47878

**Published:** 2023-10-28

**Authors:** Akinari Yamano, Mikito Hayakawa, Yoshiro Ito, Atsushi Hidano, Hisayuki Hosoo, Aiki Marushima, Eiichi Ishikawa, Yuji Matsumaru

**Affiliations:** 1 Department of Stroke and Cerebrovascular Diseases, University of Tsukuba Hospital, Tsukuba, Ibaraki, JPN; 2 Department of Neurosurgery, Institute of Medicine, University of Tsukuba, Tsukuba, Ibaraki, JPN; 3 Department of Neurology, Institute of Medicine, University of Tsukuba, Tsukuba, Ibaraki, JPN

**Keywords:** ascending pharyngeal artery, occipital artery, proatlantal artery, external carotid artery, internal carotid artery, anomalous anastomosis, anatomy, arterial embryology

## Abstract

Anomalous external carotid artery (ECA)-internal carotid artery (ICA) anastomosis is a rare variant of cervical carotid artery formation that forms an arterial ring in the cervical segment, and its embryological mechanism is still unknown. We report a case of a 41-year-old woman who was incidentally diagnosed with this arterial variation using digital subtraction angiography. The angiography revealed the occipital artery arising from the anastomotic vessel and the ascending pharyngeal artery arising from the ICA near the anastomosis. The proximal ICA was smaller in diameter than the proximal ECA, but it was not stenotic and had sufficient caliber for the distal blood flow. It is commonly believed that the persistence of primitive vessels is the result of agenesis or hypoplasia of the proximal artery. In our case, the anomalous vessel was considered to be the remnant of a primitive anastomosis between the ECA and the ICA via the pharyngo-occipital system, and the narrowing of the proximal ICA may be the result of the remaining ECA-ICA anastomosis.

## Introduction

Anomalous external carotid artery (ECA)-internal carotid artery (ICA) anastomosis is a rare anatomic variant of the carotid artery that forms an arterial ring in the cervical segment [1-5]. In previous publications, it has been hypothesized that this anomalous anastomosis is a variant of a non-bifurcating cervical carotid artery (NBCCA), which is assumed to be the result of agenesis of the ICA proximal segment [5,6]. However, its embryologic mechanism remains unclear due to the high rarity of this variation.

Here, we present a case of an anomalous ECA-ICA anastomosis diagnosed incidentally using digital subtraction angiography (DSA) and provide a brief literature review of previously published cases.

## Case presentation

A 41-year-old woman presented with a sudden-onset headache. At the outpatient clinic, she was neurologically intact and underwent brain magnetic resonance imaging as a screening test for the headache. Magnetic resonance angiography (MRA) showed an aneurysm of the anterior communicating artery (AComA) without any intracranial hemorrhage. Three months later, diagnostic cerebral angiography was performed at our hospital for a more detailed examination.

**Figure 1:**
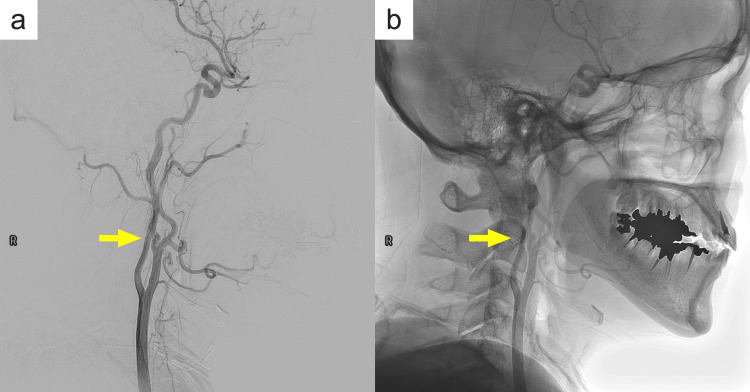
Right common carotid angiogram The lateral view on digital subtraction angiography (a) and the live image (b) of the right common carotid angiogram. The anomalous vessel connecting the internal carotid artery (ICA) and external carotid artery (ECA) at the level of the C2 vertebral body (yellow arrow). The proximal ICA is 3.2 mm and the distal ICA is 3.4 mm in diameter.

**Figure 2:**
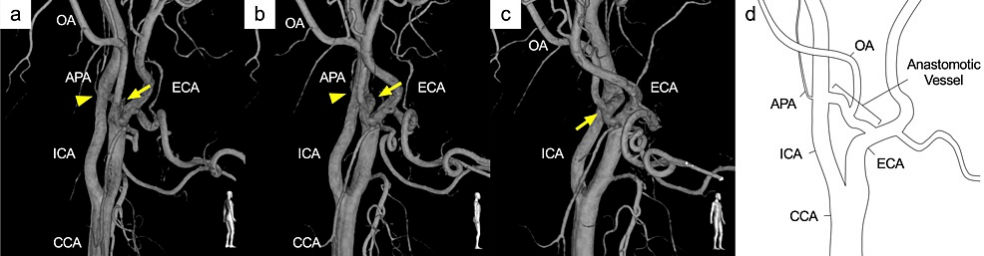
Three-dimensional images and diagram Three-dimensional rotational angiography (a-c) and the diagram (d) of the right common carotid angiogram. The occipital artery originates from the anomalous vessel (yellow arrow) and the ascending pharyngeal artery originates from the internal carotid artery in close proximity to the anomalous vessel (yellow arrowhead). APA: ascending pharyngeal artery, CCA: common carotid artery, ECA: external carotid artery, ICA: internal carotid artery, OA: occipital artery

DSA detected an AComA aneurysm 5.9 mm in diameter and incidentally revealed an anomalous ECA-ICA anastomosis on the right side at the level of the C2 vertebral body (Figure 1). Three-dimensional rotational angiography showed the occipital artery (OA) arising from the anomalous anastomotic vessel and the ascending pharyngeal artery (APA) arising from the ICA in close proximity to the anomalous vessel (Figure 2). The proximal ICA was smaller in diameter than the proximal ECA, but it was not stenotic and had sufficient caliber for the distal blood flow. On the other hand, the distal ICA had a larger diameter than the proximal ICA. The course of the cervical ICA was normal, and no other cerebrovascular anomaly was detected on examination.

## Discussion

In humans, at an early embryologic stage, the aortic arch and its branches develop from the aortic sac, the dorsal aorta, and six pairs of aortic arches. The ICA originates from the third aortic arch and dorsal aorta with regression of the ductus caroticus [7]. Lasjaunias et al. divided the ICA into seven segments based on embryonic arteries or their remnants. According to this theory, the third aortic arch is the first segment of the ICA [8,9]. Furthermore, Komiyama divided this segment of the third aortic arch into three new segments. The segments were defined by two primitive arteries arising from the third aortic arch, the primitive proatlantal artery and the primitive hypoglossal artery [10].

The anomalous ECA-ICA anastomosis, a developmental variant of the cervical carotid artery, is rare; to the best of our knowledge, only five cases of this anomaly have been reported (Table 1) [1-5]. All the cases are reported from East Asia (4 cases from Japan and 2 cases from Korea). Some genetic predispositions might be related to this anomaly, but this cannot be definitive since the number of cases is very small and this anomaly itself is not widely known. Four cases were incidentally diagnosed as this anomaly, and two cases were diagnosed after cerebral ischemic stroke. However, in both cases, the relationship between the anomaly and cerebral infarction has not been described. No age- or sex-related trends with this anomalous anastomosis were not confirmed.

**Table 1:** Characteristics of patients with external carotid artery-internal carotid artery anastomosis Characteristics of the anomalous external carotid artery (ECA)-internal carotid artery (ICA) anastomosis and imaging findings in previous reports and in our case. * The proximal ICA narrowing is calculated from the luminal diameter of the distal and proximal portions of the ICA across the anomalous vessel according to the North American Symptomatic Carotid Endarterectomy Trial method. APA: ascending pharyngeal artery, CTA: computed tomographic angiography, DSA: digital subtraction angiography, ECA: external carotid artery, ICA: internal carotid artery, MRA: magnetic resonance angiography, ND: not described, OA: occipital artery

Cases	Age/Sex	Anomalous side	Origin of the OA	Origin of the APA	The proximal ICA narrowing*	Anastomosis level	Diagnostic modality
Suzuki T, 2000 [1]	68/F	Right	ECA	ICA	20.0%	Mid-cervical segment	Cadaver
Nakai K, 2012 [2]	71/M	Right	ND	ND	29.4%	Mid-cervical segment	CTA
Uchino A, 2013 [3]	89/M	Left	ECA	ND	82.4%	Mid-cervical segment	MRA
Kim C, 2015 [4]	44/M	Left	Anastomotic vessel	ND	33.3%	C2-3	DSA
Lee B, 2020 [5]	67/M	Left	Anastomotic vessel	ECA	38.1%	C2-3	CTA
Our case, 2023	41/F	Right	Anastomotic vessel	ICA	5.9%	C2	DSA

Focusing on the anatomical features of previous reports, in all six cases, the anastomotic vessels branched at the mid-cervical segment of the ICA, and the course of the distal ICA was normal, passing through the canalis caroticus [1-5]. Because of these features, this variant differs from the remnant of the second aortic arch, which is known as an aberrant ICA; the anastomosis between the caroticotympanic artery and the inferior tympanic artery in the temporal bone [11,12]. Therefore, we hypothesize that the anastomotic vessel in the anomalous ECA-ICA anastomosis originates from the third aortic arch.

The OA and APA are thought to develop from the primitive proatlantal artery and the primitive hypoglossal artery [10]. The OA and APA both arise from the third aortic arch, sometimes share a common trunk, and rarely originate from the ICA [13-17]. In the reported cases of anomalous ECA-ICA anastomoses, the ectopic origin of the OA or APA was observed in four out of six cases, and no description of the OA or APA origin was found in the remaining two cases. In addition, Lasjaunias et al. reported a potential anastomosis between the ICA, ECA, and VA via the proatlantal artery types I and II [14]. According to their report, this anomalous ECA-ICA anastomosis is assumed to originate from the atypical remnant of the proatlantal artery, and the anastomotic vessel and the pharyngo-occipital system appear to have a close relationship, which results in the ectopic origin of the OA and APA.

We calculated the severity of the proximal ICA narrowing in previously reported anomalous ECA-ICA anastomosis cases based on the diameter of the distal and proximal ICAs according to the North American Symptomatic Carotid Endarterectomy Trial method (the percentage of vessel narrowing was measured with the ImageJ software (National Institutes of Health, Bethesda, MD), using jpeg files converted from the pdf files of the publications) [18]. In most previously published cases with anomalous ECA-ICA anastomosis, the proximal ICA was slightly patent or narrowed [1-5]. This may suggest that hypoplasia of the proximal ICA may have induced the persistence of a primitive anastomotic vessel between the ICA and ECA, similar to the occurrence of the NBCCA. In several cases of NBCCA, arterial stumps were located at the expected carotid bifurcation [4,19,20]. Such arterial stumps are presumed to be remnants of the ICA agenesis. But there are some cases, including ours, where the diameter of the proximal ICA is relatively large and its lumen is sufficient to allow good distal anterograde flow [1,2]. It is commonly believed that the persistence of primitive vessels is the result of agenesis or hypoplasia of the proximal artery [8,9], but in our case, there seems to be no need for the persistence of primitive anastomosis for sufficient distal flow. Based on these cases, the smaller diameter or the regression of the proximal ICA may be the result of a remnant of the anomalous ECA-ICA anastomosis.

These facts suggest that this variant of the anastomosis forms due to an atypical remnant of ECA-ICA connection via primitive proatlantal artery and atypical regression of the third aortic arch artery. The proximal ICA, which is relatively narrow compared to the ECA, is assumed to be a remnant of the first or second segment according to Komiyama’s concept [10]. Agenesis or hypoplasia of the proximal ICA may not be needed for such an anomalous anastomosis to remain, thus the mechanism of the development of this variant is similar but not necessarily the same as in the case of the NBCCA.

## Conclusions

We reported a rare case of anomalous ECA-ICA anastomosis originating from the ectopic OA and APA. This anastomotic variant suggests a potential connection between the cervical ECA and ICA via the proatlantal artery. The remaining ECA-ICA anastomotic vessel in this rare variant may not be the result of atypical agenesis or hypoplasia of the proximal ICA. Although this anomaly is rare, it should be known to avoid complications during cervical vascular surgery and neuroendovascular treatment.
